# Brainstem Encephalitis as an Atypical Neurologic Complication Following Kikuchi–Fujimoto Disease

**DOI:** 10.1155/crnm/3086387

**Published:** 2025-07-14

**Authors:** Youjiang Tan, Tyngyu Chuah

**Affiliations:** ^1^Department of Neurology, Singapore General Hospital, Singapore; ^2^Department of Rheumatology, Sengkang General Hospital, Singapore

## Abstract

We report an uncommon and peculiar case of a patient who developed brainstem encephalitis between three and four months after recovering from an episode of Kikuchi–Fujimoto disease (KFD). She presented acutely with oscillopsia and persistent irrepressible hiccups, for which brainstem stroke was initially suspected. Brain magnetic resonance imaging was negative for ischemic strokes but demonstrated an enhancing T2-hyperintense lesion within the area postrema of the medulla oblongata extending into the upper cervical cord. Workup for infections etiologies and demyelinating disorders of the central nervous system including neuromyelitis optica, multiple sclerosis, and antimyelin oligodendrocyte glycoprotein antibody disease, were unremarkable. Prior to the administration of immunosuppressive treatment, she spontaneously and rapidly recovered, remaining well over a 3-year period of follow-up. We reviewed prevailing scientific literature and identified similar, albeit rare, cases of encephalitis which were attributed to KFD, which we added to our discussion.

## 1. Introduction

Kikuchi–Fujimoto disease (KFD) is a rare yet self-limiting histiocytic necrotizing lymphadenitis of unknown etiology, characterized by painful lymphadenopathy, cutaneous manifestations, and systemic features of fever, arthralgia, and malaise [1]. Neurological complications are uncommon, and delayed presentations of encephalitis have been reported sporadically in medical literature [1–6]. In this report, we describe the case of a middle-aged woman who developed brainstem encephalitis 3 months after recovering from KFD, which was attributed to her preceding KFD after extensive investigations and tests.

## 2. Case Description

A forty-three-year-old woman first presented with fever and multiple painless but enlarged cervical nodes. Extensive investigations revealed anemia (hemoglobin 9.6 × 10^9^/L; normal [N]: 12–16 × 10^9^/L), leukopenia (white cell count 1.46 × 10^9^/L; N: 4–10 × 10^9^/L), and abnormal inflammatory markers with elevated C-reactive protein (43.6 mg/L; N: < 3 mg/L) and lactate dehydrogenase levels (526U/L; N: 3.9–6.0 U/L), and an increased erythrocyte sedimentation rate (38 mm/h; N: 1–10 mm/h). Blood work for infective etiologies returned negative. However, her cervical lymph node biopsy revealed geographical paracortical lymphohistiocytic necrosis with abundant karyorrhectic debris extending into the follicular regions. Features suggestive of malignancy were absent, and nucleic acid amplification and culture for *Mycobacterium tuberculosis* along with bacterial and fungal cultures of the biopsy specimens yielded unremarkable results. With clinicopathologic features consistent with KFD, she received a short course of oral prednisolone, and her lymphadenitis resolved and her inflammatory markers normalized thereafter.

**Figure 1:**
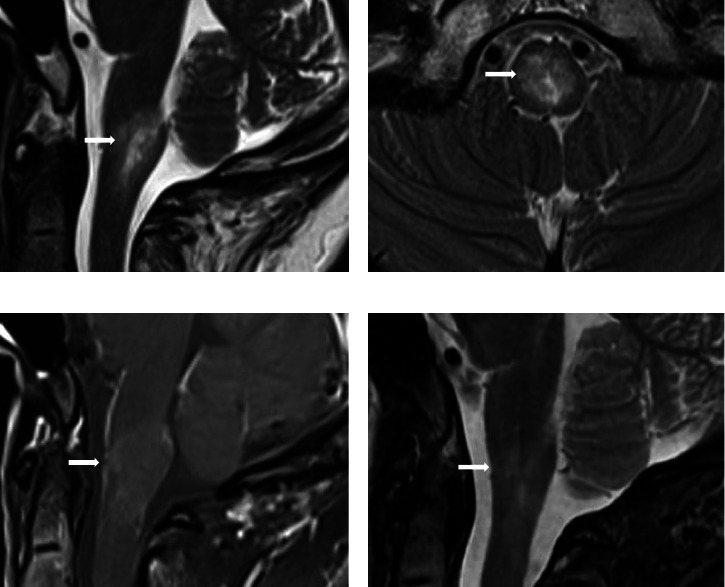
Magnetic resonance imaging (MRI) of the brain. In the first MRI performed to investigate her persistent hiccups and upbeating nystagmus, T2- (a-b) and T1-weighted (c) projections revealed a T2-hyperintense intra-axial lesion in the lower medulla and upper cervical cord (white arrow) which enhances peripherally with gadolinium (c; white arrow). Sagittal T2 projection of another MRI performed 4 months later showed a reduction in the extent of the lesion (d; white arrow).

Three months later, she presented acutely with oscillopsia and persistent irrepressible hiccups. Importantly, the typical symptoms of KFD did not recur. Neurological examination showed the presence of upbeating nystagmus, but concomitant sensorimotor deficits and cranial nerve palsies were absent. Suspecting brainstem stroke, an urgent computed tomography scan of her brain showed no radiological abnormality, but a brain magnetic resonance imaging (MRI) with gadolinium contrast performed soon after revealed a T2-hyperintense lesion within the area postrema of the medulla oblongata which extended into the upper cervical spine (Figures 1(a), 1(b), and 1(c)). Cervicothoracic MRI demonstrated no additional abnormalities involving the spinal cord. Cerebrospinal fluid (CSF) examination and microbiological tests were unremarkable, and oligoclonal bands were absent. Serological tests for antibodies against aquaporin-4 and myelin oligodendrocyte glycoprotein, as well as the antinuclear antibody (ANA) test and extractable nuclear antigen (ENA) profile, were all negative. While the aforementioned investigations were being performed, her hiccups and nystagmus spontaneously and rapidly improved within days from onset, prior to the initiation of any treatment. Despite being without immunosuppressive/immunomodulatory treatment, a follow-up brain MRI 4 months later showed remarkable radiological improvement, and she remained well over the next 3 years (Figure 1(d)).

## 3. Discussion

Neurological complications of KFD are uncommon and varied, posing significant diagnostic challenges to physicians [1–4]. Encephalitis in particular is exceedingly rare, with less than 10 cases being reported worldwide, most occurring concurrently or soon after the onset of typical KFD symptoms [3, 4]. Despite the absence of a brain biopsy due to her rapid recovery, we attributed our patient's brainstem encephalitis to KFD as the self-limiting nature of her clinicoradiological features was typical of that condition [2–5]. This was further supported by the lack of other likelier diagnoses despite our extensive investigations. Moreover, her case closely resembled an earlier report by Shabana et al. describing a woman with KFD-related encephalitis who developed left arm weakness 3 months after the onset of typical KFD symptoms, with brain MRI subsequently showing enhancing T2-hyperintense abnormalities in her right basal ganglia and left caudate head. [5]. This time interval between the onset of KFD symptoms and neurologic deficits, during which typical KFD symptoms resolved completely in both patients, suggests that their encephalitis may be an atypical manner by which KFD recurs or relapses. Furthermore, their rapid and spontaneous clinicoradiologic improvement in the absence of immunosuppressive treatment hints at a similarly transient, though yet fully understood, inflammatory process characteristic of KFD.

Our report adds on to the current knowledge of KFD-related neurologic complications. First, encephalitis can be a late complication of KFD, and both the neurologists and rheumatologists should, therefore, be aware of this uncommon but important condition when patients present with acute neurological deficits up to as long as 3 months after recovery from KFD. Moreover, the patients' neurological presentations can falsely resemble more common neurological disorders such as ischemic strokes. In such situations, these patients risk being inappropriately treated with thrombolysis and/or antithrombotic treatment, potentially exposing them to treatment-related complications. Second, clinical prognosis of KFD-related encephalitis appears excellent. A recent study reviewing previous reports on limbic encephalitis found a good clinical response to corticosteroid treatment [6]. Furthermore, clinical recovery appears sustained in reports of adults (including ours) who developed encephalitis after KFD, even when without maintenance immunosuppressive treatment [5, 6]. Longitudinal observation alone after discharge is, therefore, reasonable, sparing patients from the complications and side effects of long-term immunosuppression. However, the exact pathogenic processes leading to their encephalitis, and why the area postrema was specifically involved in our patient, are questions which cannot be answered presently and awaits further elucidation as more cases are identified and reported in the future.

## Data Availability

Research data are not shared.
